# Climate change and the health of older adults in Europe: a call for a geriatric climate medicine framework

**DOI:** 10.1007/s41999-025-01336-3

**Published:** 2025-10-22

**Authors:** Isabel Lozano-Montoya, Claudia Ruiz-Huerta, Francisco J. Gómez-Pavón

**Affiliations:** 1 Servicio de Geriatría, Hospital Universitario Central de La Cruz Roja San José y Santa Adela, C/Reina Victoria, 24, 28003 Madrid, Spain; 2 Facultad de Medicina, Universidad Alfonso X El Sabio, Avda. de La Universidad, 1, , 28691 Villanueva de La Cañada, Madrid, Spain; 3 Servicio de Medicina Preventiva, Hospital Universitario Central de La Cruz Roja San José y Santa Adela, C/Reina Victoria, 24, 28003 Madrid, Spain

**Keywords:** Climate crisis, Geriatric climate medicine, Older adults, Heatwaves, Adaptation, Frailty

## Abstract

**Purpose:**

Climate change is a critical determinant of health that disproportionately affects older adults. This review synthesises recent evidence on climate-related health risks in older Europeans and proposes a geriatric climate medicine framework to guide clinical practice, health system preparedness and policy action.

**Methods:**

Narrative review of scientific literature and policy documents published between 2019 and 2025, with emphasis on European epidemiological data and adaptation frameworks. Studies were included if they reported health impacts or adaptation/mitigation strategies relevant to adults aged ≥ 65 years.

**Results:**

Heatwaves, chronic and acute exposure to air pollutants, flooding and the expanding range of climate-sensitive infectious diseases increase hospitalisation, cognitive decline, and mortality in older adults, especially in women, those aged ≥ 80 years, and individuals with comorbidities or frailty. These risks remain insufficiently addressed in geriatric clinical practice and health policy.

**Conclusions:**

In a Europe warming at twice the global rate, urgent integration of climate risk assessment into geriatric care, enhanced resilience of health and social care infrastructure and climate policies that prioritise older adults are essential to reduce inequities and improve health outcomes.

## Key summary points

**Aim:**

To propose a European geriatric climate medicine framework integrating climate risk assessment into clinical practice, health system preparedness and policy advocacy.

**Findings:**

Heatwaves, air pollution, flooding, and emerging infectious diseases increase morbidity and mortality in older adults, particularly those living with frailty, yet remain under-represented in clinical guidelines and European policies.

**Message:**

European geriatrics should lead the integration of climate risk into clinical care, strengthen the resilience of hospitals and long-term care facilities, and promote inclusive policies that prioritise equity and prevention.

## Introduction

Climate change is a major public health threat, driven by rising temperatures, more frequent extreme weather events, deteriorating air quality, and ecological disruption. Older adults are disproportionately affected due to age-related physiological changes (including impaired thermoregulation, multimorbidity, polypharmacy, and functional decline) and reduced adaptive capacity from social and economic factors, such as isolation, inadequate housing, and limited access to care [1–3].

Europe is warming at approximately twice the global average rate, amplifying the frequency and severity of climate-related hazards [2]. Heatwaves, chronic air pollution, flooding, and the emergence of climate-sensitive infectious diseases contribute to increased morbidity, hospitalisation, and mortality in older adults, particularly those living with frailty [3]. Despite these growing risks, climate change remains under-represented in geriatric clinical guidelines and in the design of health and social care systems.

While some European public health strategies address climate-related health threats, few explicitly target the needs of older populations. To our knowledge, no comprehensive European framework currently integrates climate risk into geriatric medicine and aligns clinical practice, environmental health science, and policy.

This narrative review synthesises recent evidence on climate-related health risks in older adults, highlights adaptation strategies relevant to clinical and community care, and situates them within current European policy directions. Our objective is to lay the foundation for a European geriatric climate medicine framework to drive the transition from reactive care to proactive, equitable, and climate-resilient health systems.

### Methods

This narrative review synthesises scientific and policy literature on climate-related health risks in older adults, with a focus on the European context. A non-systematic search was conducted in PubMed from January 1, 2019, to February 15, 2025. Search terms combined Medical Subject Headings (MeSH) and free-text keywords, including: “Climate Change”, “Aged” OR “Older adults”, “Heatwaves” OR “Extreme heat”, “Air Pollution”, “Floods”, “Infectious diseases”, “Climate adaptation”, “Mitigation”, and “Europe”.

Two authors independently screened titles and abstracts to identify relevant studies. Disagreements were resolved by a third author. Studies and policy reports were included if they met at least one of the following criteria: reported climate-related health impacts in populations aged ≥ 65 years or described adaptation or mitigation strategies relevant to older adults in Europe.

No restrictions were applied regarding study design. However, priority was given to peer-reviewed research, large epidemiological studies, systematic reviews, and recent European policy documents. Given the narrative nature of this review, no formal quality assessment was performed.

Evidence was synthesised under thematic domains: heatwaves, air pollution, flooding, climate-sensitive infectious diseases, health system and policy adaptation. Knowledge gaps and implications for geriatric clinical practice and European policy were also identified.

## Results

### Scientific evidence

Robust scientific evidence has consistently shown that older adults are susceptible to climate-related health impacts.

### Heatwaves: health impacts in older adults

Europe is warming at roughly twice the global average, and this accelerated trend has intensified the frequency, duration, and severity of heatwaves [2]. The health consequences for older adults are substantial. During the unprecedented summers of 2022 and 2023, heat was estimated to have caused approximately 60,000 and 48,000 excess deaths, respectively. Mortality rates were highest in southern European countries, including Greece, Bulgaria, Italy, Spain, Cyprus, and Portugal [1, 2].

Risk is not evenly distributed within the older population. Women had a higher mortality burden (female-to-male ratio 1.55), and individuals aged 80 years or more faced mortality rates almost nine times greater than those aged 65–79 years [4]. Pre-existing cardiovascular, respiratory, renal, or metabolic diseases amplify susceptibility, especially in those with frailty [5, 6].

Beyond mortality, heatwaves are linked to a rise in acute morbidity. Studies confirm increases in cardiovascular events through mechanisms, such as autonomic dysfunction, systemic inflammation, and impaired thermoregulation. Hospital admissions also rise among people with Alzheimer’s disease and related dementias during extreme heat, underscoring their vulnerability [7, 8]. These observations emphasise the need for timely, region-specific public health measures designed with older adults in mind.

### Air pollution: chronic exposure and cognitive decline

Long-term exposure to fine particulate matter (PM₂.₅) remains a significant environmental hazard for older adults in Europe. Chronic inhalation of PM₂.₅ has been associated with increased cardiovascular and respiratory mortality, as well as a higher prevalence of frailty [9]. In a WHO-commissioned meta-analysis, Orellano et al. confirmed elevated risks for both cardiovascular and respiratory deaths in adults aged 65 years and older [10].

The neurological consequences are increasingly recognised. In England, Wood et al. reported that sustained exposure to PM₂.₅ and nitrogen dioxide (NO₂) was linked to accelerated decline in memory and executive function. These associations persisted after adjusting for sociodemographic and health-related factors, suggesting a direct, independent effect [11].

Acute episodes of poor air quality can also exacerbate existing conditions, triggering hospital admissions for cardiovascular and respiratory disease. Given the cumulative evidence, reducing ambient air pollution should be considered a public health priority with clear co-benefits for healthy ageing.

### Wildfires: heat and smoke risks for older adults

The summer of 2025 has seen an unprecedented wildfire season across Europe. In southern France’s Aude region, a wildfire burned over 17,000 hectares, marking the largest wildfire in nearly 75 years. At the same time, Spain, Portugal and Greece are all suffering extensive fires due to record-breaking heatwaves. The EU Science Hub reports over 409,000 hectares burned so far in 2025, nearly double the 19-year average [12].

Older adults are at risk due to combined exposure to extreme heat, smoke, and air pollutants, such as fine particulate matter (PM₂.₅) [1, 2]. Evidence highlights increased hospitalisations for cardiovascular and respiratory disease, exacerbations of chronic obstructive pulmonary disease, and heat-related illnesses in older populations during wildfire events [1, 5].

The impacts extend beyond the acute phase. Displacement, disruption of medical care, and loss of social support can exacerbate frailty, and contribute to functional and mental decline [1, 5, 8]. Adaptation strategies should integrate wildfire preparedness into geriatric care plans, including smoke advisories, ensuring medication supply, and prioritisation of older adults in evacuation protocols [5].

### Climate-sensitive infectious diseases: expanding threats in Europe

Rising temperatures, altered precipitation patterns, more frequent extreme weather events and changes in land use are increasing the environmental suitability for the transmission of multiple vector- and waterborne pathogens, expanding both their geographic distribution and seasonal activity [1, 2].

West Nile virus (WNV) is a prominent example: it has shown a 256% increase in outbreak risk since the 1950s, with severe neuroinvasive forms affecting older adults. France, Italy, and Spain have reported autochthonous transmission of dengue, driven by the spread of Aedes albopictus. Climatic suitability for leishmaniasis has increased particularly in southern Europe, while northern coastal regions are facing a rising risk of Vibrio infections, which constitute a serious concern for older adults with underlying health conditions. Tick-borne diseases such as Lyme disease and tick-borne encephalitis (TBE) are also expanding beyond their historical endemic areas, with prolonged tick activity linked to milder winters [1, 2, 13].

The clinical implications for older populations are substantial. Both WNV and TBE can cause severe neurological disease, while Vibrio infections often present with rapidly progressive sepsis [1, 2]. Gaps in clinician awareness persist, and many adaptation plans do not include adequate systems for disease surveillance, diagnostic capacity, or targeted public health messaging to address these emerging threats in older adults.

### Flooding: infectious disease risks and health service disruption

Extreme precipitation events are becoming more frequent worldwide, driving a rising risk of floods. Between 2014 and 2023, more than 60% of global land areas experienced intensified extreme rainfall [Romanello]. Flooding poses both acute and long-term threats to the health of older adults.

The catastrophic floods that devastated Valencia, Spain, in October 2024 illustrate the multifactorial risks associated with extreme weather events. These floods caused more than 200 deaths and widespread disruption of healthcare services. Over half of the region’s primary care centres were affected, and access to electricity, gas, and clean water was severely compromised [14]. The Spanish Ministry of Health identified a moderate risk of infectious diseases (including leptospirosis, legionellosis, and gastroenteritis) particularly among older adults and individuals with chronic illnesses [15].

Older adults face specific challenges during floods due to sensory impairments, functional decline, and social isolation, all of which can compromise timely receipt of alerts or safe evacuation. Interruptions to medical care and social support can exacerbate existing medical conditions and increase the likelihood of adverse physical and mental health outcomes [14, 15].

Severe flooding events also occurred across Europe in 2025, including in southern France, Italy (Emilia-Romagna, Tuscany, Piedmont), the United Kingdom (Greater Manchester, Leicestershire), and Romania. Together, these events underscore the urgency of prevention and response strategies that explicitly prioritise the needs of older adults from early warning systems to rapid restoration of essential services.

### Food and water insecurity: nutritional and hydration challenges

Extreme weather events, drought, and heatwaves are contributing to regional food insecurity and involuntary displacement. Climate-related conditions caused food insecurity in nearly 12 million additional Europeans in 2021 [2]. Functional decline and limited access to support networks make older adults (especially those living alone or in low-income settings) more vulnerable.

### Climate-related stressors: implications for mental health

Beyond physical health impacts, climate-related stressors also have significant consequences for cognitive and mental health. Heatwaves have been associated with increased stroke incidence, delirium, and acute exacerbations of dementia in older adults. [8, 16, 17]. Furthermore, climate disasters such as floods and wildfires can trigger anxiety, depression, post-traumatic stress and isolation in older adults [18].

### Adapting geriatric care to a changing climate

Adapting geriatric care to climate change requires action across clinical, institutional, and policy domains. The following framework outlines key strategies to reduce vulnerability and enhance resilience in older adults.

### Individual-level care and clinical practice adaptations

Integrating climate risk into routine geriatric care begins with the proactive identification of frail older adults and those with cognitive or functional decline who are most vulnerable to extreme weather events [5, 16]. Clinicians should provide targeted heat-health counselling: adequate hydration, light and breathable clothing, maintaining a cool environment, and recognising early signs of heat-related illness [19]. Medication reviews are essential during hot periods, particularly for drugs that impair thermoregulation (such as diuretics, anticholinergics, or psychotropics) with adjustments made when clinically appropriate [5, 19].

Where air conditioning is not available, practical alternatives include cool baths, shading, or the use of fans combined with skin wetting (bearing in mind that fans lose effectiveness above 35 °C). Referrals to cooling centres should be arranged for high-risk patients, ensuring transport support, where functional impairment exists [5]. During pollution episodes or wildfire smoke events, older adults with cardiovascular or respiratory disease should remain indoors with effective air filtration and use high-efficiency masks when outdoor exposure is unavoidable [2, 5].

For individuals with dementia, personalised disaster preparedness plans should incorporate caregiver oversight to ensure safety [5, 19]. The implementation of teleconsultations or home visits can help ensure continuity of care while minimising exposure risks [5, 19]. Climate-informed geriatric care requires proactive, individualised, and socially supported interventions.

### Long-term care and community support measures

In long-term care facilities and community-based services, adaptation measures must be coordinated and systematic [5]. Care homes should maintain reliable cooling systems with backup power, designate temperature-controlled rooms, and ensure staff are trained to detect early signs of heat stress and dehydration [5]. Evacuation protocols for wildfire or flood events must include specific provisions for patients with dementia or functional decline [2, 5]. Regular emergency drills with the involvement of families can improve the resilience of nursing homes [19].

At the community level, municipalities should guarantee access to cooling centres, especially for individuals with functional impairment. During extreme weather events, home care teams and social services should conduct wellness checks, deliver cooling devices, and ensure the provision of essential supplies. Addressing social isolation is also critical: local services must proactively identify older adults living alone and connect them with community support networks [1, 2, 5, 19].

Emergency services can maintain registries of individuals reliant on electrically powered medical equipment, enabling prioritisation during outages or evacuations [2, 15, 19]. Coordinated action between care institutions, community programmes, and municipal services is essential to building local resilience and protecting older adults from climate-related hazards [2, 5].

Older adults may also face specific barriers to participating in climate adaptation measures, including the high cost of green technologies (which can limit the uptake of solutions, such as solar panels or electric vehicles) and the digital divide, which restricts access to online information and management tools [20].

### Policy-level interventions

Effective adaptation of geriatric care to climate change requires coordinated action across the entire health system, supported by policies that explicitly address the needs of older adults [2, 5, 21]. Climate adaptation planning within health systems should incorporate geriatrics into every stage, from risk assessment to response, ensuring that early warning systems trigger targeted measures, such as heat-health alerts, proactive home visits, and telephone check-ins for vulnerable individuals [5, 19]. Standardised training for health professionals is essential to strengthen preparedness [5, 7].

Infrastructure resilience is another priority. Investments in emergency power generation, secure water supply, and passive cooling design can help maintain continuity of care during extreme events [19]. Public policies should also support home adaptations, such as installation of air conditioning or improved insulation [2, 19].

Urban planning plays a key role, with the expansion of green areas especially in high-temperature areas that leads to both environmental and health benefits. Climate strategies at local, national, and European levels must include older adults in risk assessments, adaptation programmes, and disaster response plans [2, 5].

The health-care sector in the European Union is itself a significant contributor to climate change, accounting for approximately 5% of total greenhouse gas emissions. Integrating mitigation strategies, such as improving energy efficiency in facilities, prioritising reusable over single-use medical equipment where clinically appropriate, and applying environmental standards to the procurement of external services (including cleaning, catering, and laundry) can reduce the sector’s carbon footprint [22]. These measures not only support climate goals but also enhance the long-term sustainability and resilience of health services.

Aligning geriatric care, public health, and policy initiatives will be essential to achieve equitable and climate-resilient health systems [2, 21, 22].

### Geriatric leadership

Geriatricians are uniquely positioned to lead multidisciplinary strategies that protect older adults from the health impacts of climate change, especially extreme weather events [21]. These strategies must integrate clinical care with social support. By collaborating with public agencies, health departments, and social services, geriatricians can contribute to the design of structured heat-health action plans, emergency preparedness protocols, and equitable adaptation measures, such as early warning systems and housing interventions [2, 19].

In long-term care facilities, geriatricians can guide the development of institution-specific protocols and staff training programmes for the early detection and management of heat-related illness [19]. They also play a key role in promoting sustainable health care practices, recognising that the health-care sector contributes significantly to greenhouse gas emissions. Initiatives such as expanding telemedicine, supporting deprescribing when clinically appropriate, and favouring non-invasive management strategies can reduce environmental impact while aligning with patient-centred care [1, 22].

Drawing on their interdisciplinary perspective and commitment to person-centred care, geriatricians can drive the implementation of climate-resilient interventions across health systems, ensuring that older adults are prioritised in both adaptation and mitigation strategies [1, 2].

### European geriatric climate medicine framework

In recent years, a growing number of European policies and programmes have begun to acknowledge the vulnerability of older adults to climate change. The EU Adaptation Strategy, for example, encourages Member States to integrate climate risks into health planning, including the development of heat-health action plans [23]. Countries such as Spain, France, and Germany have already introduced alert systems and targeted measures for older adults [15]. Platforms such as Climate-ADAPT and the European Climate and Health Observatory provide data and tools to help identify at-risk older adults and to support the design of age-sensitive adaptation measures [24]. While many of these initiatives are not designed exclusively for this age group, there is increasing recognition of their needs in emergency preparedness, healthcare infrastructure planning, and urban resilience strategies.

**Fig. 1:**
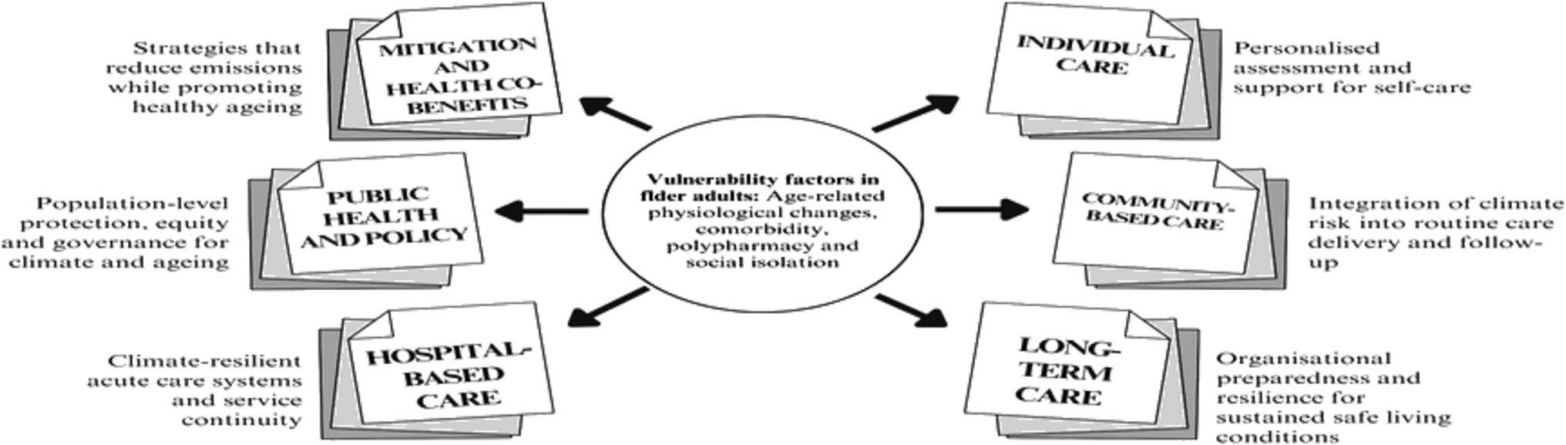
European geriatric climate medicine framework outlining seven strategic domains for integrating climate resilience into geriatric health systems, with a central focus on vulnerability factors in older adults

Building on these foundations, we present the European geriatric climate medicine framework (Fig. 1). This conceptual model brings together six strategic domains from individual care to mitigation measures. It is intended as a practical guide for clinicians, care institutions, and policymakers working to develop age-inclusive, climate-resilient health systems across Europe.

The framework can be implemented gradually, starting with targeted clinical protocols and then expanding towards a more comprehensive approach. Its impact can be assessed using indicators, such as reductions in heat-related hospital admissions, continuity of care during extreme events, and improvements in preparedness scores in long-term care facilities. Incorporating these indicators within national and regional adaptation plans will enable countries to monitor progress, identify gaps, and share best practices across Europe.

Table 1 provides practical recommendations for each domain of the framework, structured by level of care.

**Table 1:** Recommendations to mitigate climate change impacts in older adults

Level of Care	Recommendation	Implementation Example	
Community-based care	Incorporate climate-related risk into the clinical history	Include standardized questions in the electronic health record regarding housing conditions, access to air conditioning, social isolation and evacuation capacity	Promote “green” lifestyles, including locally sourced and seasonal nutrition, adapted physical activity and social interactions
	Identify older adults at risk of dehydration (e.g., those without air conditioning or on diuretics) during routine home assessments	Measure indoor temperature. Use electronic health records to identify high-risk individuals for proactive follow-up	
	Provide education to patients and caregivers on how to stay safe during periods of extreme heat	Distribute printed or digital materials outlining hydration strategies, cooling options and early signs of heat-related illness	
	Maintain adequate hydration and review the use of medications that may increase vulnerability to dehydration	Conduct seasonal medication reviews, provide tailored advice on fluid intake during hot months	
	Address climate-related mental health symptoms	Assess for sleep disturbances, anxiety, or cognitive decline potentially exacerbated by extreme heat and provide mental health support	
Hospital-based care	Incorporate climate-sensitive screening into clinical protocols	Screen for signs of dehydration, orthostatic hypotension or confusion during triage (especially in patients with cognitive impairment)	
	Review and adjust medications that may increase the risk of heat-related illness	Temporarily modify or suspend diuretics, anticholinergics or other high-risk drugs during heatwaves	
	Adjust healthcare delivery practices to minimise exposure during extreme events	Prioritize telemedicine and home visits for frail patients	
	Prioritize reusable medical equipment and sustainable service contracts	Select suppliers that meet energy efficiency criteria in cleaning, laundry and waste management	
Long-term care	Establish protocols for managing heat emergencies	Develop institutional action plans, including temperature monitoring, hydration rounds and adjusted activity schedules	
	Train staff in recognizing heat-related illness	Include mandatory training modules on heat stress in annual education plans	
	Promote awareness of the need for heat-resilient housing	Recommend funding applications for passive cooling interventions (e.g., external shading and improved ventilation)	
	Include environmental risk factors in routine geriatric assessments	Add specific questions on thermal comfort and structural housing quality	

### Strengths and limitations

This narrative review provides an integrated synthesis of evidence on climate-related health risks in older adults and proposes a structured framework for geriatric climate medicine, combining recent scientific findings with policy sources. The use of a non-systematic search strategy and the absence of formal quality appraisal may have resulted in the omission of relevant studies, and the fast-evolving nature of the topic may limit the longevity of the evidence base. These limitations underscore the urgency of developing a coordinated response to safeguard the health of ageing populations in a changing climate.

### Conclusion and call to action: from conceptual framework to a European agenda for geriatric climate medicine

The effects of climate change on older adults’ health are no longer theoretical; they are happening here and now. This review has synthesised current evidence on the health impacts of climate change in older adults and has proposed the European geriatric climate medicine framework as a structured model to guide age-sensitive adaptation and mitigation strategies. Turning this framework into meaningful progress requires a coordinated European agenda with clear priorities in research, education, and policy.

### Research

There is an urgent need to strengthen the scientific evidence on how climate change influences aging trajectories and chronic disease exacerbation. Understanding how environmental stressors (such as heatwaves, air pollution, and flooding) interact and contribute to the development of geriatric syndromes is crucial. Future research should also explore the effectiveness of targeted adaptation interventions for older populations across different care settings. Based on this evidence, clinicians and guideline developers should create evidence-based clinical guidelines that integrate climate-related health risks into geriatric assessment and care planning, and systematically integrate adaptation measures into routine clinical practice.

### Education

Climate literacy should become an integral component of geriatric medical training to ensure that future clinicians are prepared to recognise, manage, and mitigate climate-related health risks in older adults across both acute and long-term care settings. Furthermore, educational initiatives targeting patients and caregivers can increase awareness of climate hazards and strengthen resilience through the adoption of adaptive behaviours.

### Policy engagement interventions

Geriatricians have a critical role to play in health policy. This expertise is essential to ensure that national and European adaptation plans are age-inclusive, equity-focused and responsive to the specific needs of older adults.

### Call to action

The European geriatric community led by the European Geriatric Medicine Society (EUGMS) in collaboration with national societies should provide leadership and strategic coordination to operationalise the proposed framework, integrating climate resilience into research, education, and policy. Priorities include integrating climate risk into geriatric care, preparing health and social care systems for extreme events, and ensuring adaptation plans address the specific needs of older adults. These measures must be implemented without delay to reduce avoidable morbidity and mortality, address inequities, and harness co-benefits for health, equity, and sustainability. *The time to act is now.*
